# Awareness and attitudes toward epilepsy among medical students and interns in Riyadh, Saudi Arabia

**DOI:** 10.1017/S1463423622000597

**Published:** 2022-11-11

**Authors:** Khalid A. Bin Abdulrahman, Majed Ghanem Alharbi, Abdulmajeed Mansour Alzeer

**Affiliations:** 1 Department of Medical Education, College of Medicine, Imam Mohammad Ibn Saud Islamic University (IMSIU), Riyadh, Saudi Arabia; 2 College of Medicine, Imam Mohammad Ibn Saud Islamic University (IMSIU), Riyadh, Saudi Arabia

**Keywords:** epilepsy, seizures, medical students, Saudi Arabia

## Abstract

**Background:**

Epilepsy is a common neurological condition. It affects around 1% of the global population. This study aims to evaluate the knowledge and attitudes toward epilepsy.

**Methods:**

This is a cross-sectional observational study. An online questionnaire was distributed to medical students in their clinical and preclinical years and interns at Riyadh’s four public universities. Then a comparison was made to see whether attending more courses in medical school would influence the students’ knowledge and attitudes.

**Results:**

In the present study, 95% of medical students had heard about epilepsy or convulsive seizures (a significantly larger proportion of clinical students had heard about epilepsy than preclinical students (99.0% versus 92%, *P*-value = 0.000)). Furthermore, 34.0% believed that epilepsy could be treated. Moreover, 79.1% of those polled claimed that brain disease originated from epilepsy, followed by genetic factors (64.1%) and convulsions (92.3%) as the most common symptoms.

**Conclusion:**

Regarding medical students’ awareness of epilepsy, it turns out that it is good and better than reported in other research, especially among clinical students rather than preclinical students, who have a negative attitude toward epileptic patients. Consequently, there is a need to further development of their knowledge throughout future campaigns and conferences, and curricula that should be tailored to help improve awareness and attitudes toward epilepsy.

## Introduction

Approximately 80% of epilepsy patients live in developing countries where epilepsy remains a significant public health problem due to its health and social, cultural, psychological, and economic consequences (Njamnshi *et al.* 2010; Yemadje *et al.* 2011). It is well known that epilepsy patients face social discrimination based on negative social attitudes and misconceptions. This discrimination against patients with epilepsy may be attributed to a lack of knowledge and understanding of epilepsy (Aydemir, 2011). Misconceptions and beliefs about epilepsy have serious negative social and psychological consequences for people with epilepsy, including fear, humiliation, and restrictions on social interactions. Stigma and discrimination are two of the most significant challenges to the optimal management of epilepsy (Daoud *et al.* 2007; Tuan *et al.* 2007).

Epilepsy has many psychological and social consequences due to ignorance and misconceptions about epilepsy in the population, resulting in stigmatization and isolation of patients with epilepsy. Therefore, signs of depression may be observed (Hirfanoglu *et al.* 2009). The attitude of family members might influence epilepsy. For example, overprotective behavior and low expectations of academic and social performance can negatively impact each patient (Rose, Peace & McBride, 1955; Jacoby *et al.* 2004; Caixeta *et al.* 2007; Hirfanoglu *et al.* 2009).

Knowledge about epilepsy will influence their interactions with people with the disorder and thus affect public perceptions of people with it. Furthermore, knowledge of epilepsy among medical students is rarely assessed in the literature. This study aims to report on epilepsy and attitudes and practices regarding patients with epilepsy in Riyadh, Saudi Arabia.

## Methods

This is a cross-sectional observational study using an online-based questionnaire distributed among medical students in their clinical and preclinical years and interns in the four public universities in Riyadh, Saudi Arabia. These include Imam Mohammed Ibn Saud Islamic University, King Saud University, King Saud bin Abdulaziz University for Health Sciences, and Princess Nourah University. All participants provided their informed written consent to participate in the study. The study was carried out according to the Declaration of Helsinki and was approved by the Institutional Review Board of the IRB Committee of Imam Mohammad Ibn Saud Islamic University, number 120–2020 dated Dec 08, 2020.

The knowledge about epilepsy was compared to see if taking more courses in medical school would influence their awareness and attitudes. A standardized questionnaire consists of 23 questions (Alaqeel *et al.* 2013; Alomar *et al.* 2020).

To obtain reliable results, the study sample size was estimated to be around 383+ participants, with a confidence level of 95% that the real value is within ±5% of the measured/surveyed value. They were calculated using calculator.net/sample-size-calculator. (Anon, n.d.). The inclusion criteria included all medical students and interns who study at the four public universities in Riyadh city. On the contrary, the exclusion criteria included (1) nonmedical student, (2) medical students studying outside Riyadh, and (3) respondents who did not consent to participate in the study. Following the questionnaire distribution, MS Excel was used for data entry, while SPSS version 23 was used for data analysis. Frequency and percentage were used for categorical variables. Chi and *t*-tests were used to determine factors affecting students’ knowledge and attitudes. All statements were considered significant if the *P*-value was lower than or equal to 0.05.

## Results

In this study, 1039 responses to our questionnaire were collected. Most of the responses came from Imam Mohammad Ibn Saud Islamic University (IMSIU) (40.7%), while 27.3% came from King Saud University for Health Sciences and 24.3% from Princess Nourah University. Furthermore, most of the participants were between 20 and 25 years old (83.3%), and almost all were single (97.0%). Considering the academic year, 54.7% of the students were in preclinical years (Table 1).

**Table 1.** Demographic factors of participants (*N* = 1039)

		Count	Column *N* %
Which university do you study in?	King Saud University	80	7.6%
	King Saud University for health sciences	286	27.3%
	Al-Imam Mohammad Ibn Saud Islamic University	427	40.7%
	Princess Nourah University	255	24.3%
Age	16–20	131	12.5%
	20–25	873	83.3%
	25–30	44	4.2%
Marital status	Single	1017	97.0%
	Married	31	3.0%
	Divorced	0	0.0%
Academic year	First year	145	13.8%
	Second year	196	18.7%
	Third year	260	24.8%
	Fourth year	199	19.0%
	Fifth year	199	19.0%
	Intern	49	4.7%
Are you currently a:	Preclinical	573	54.7%
	Clinical	421	40.2%
	Intern	54	5.2%

## Awareness

In general, 95% of medical students had heard of epilepsy or convulsive seizures, and 38.2% knew someone with epilepsy. When the results of the preclinical and clinical students were compared, it was revealed that clinical students had a significantly higher proportion of hearing about epilepsy than the preclinical students (99.0% versus 92%, *P* value = 0.000). Furthermore, 27.7% of the students would oppose their children from playing with other children with seizures, while 55.3% would not forbid their children from playing with patients with seizures. This refusal attitude was significantly higher in clinical and preclinical students (33.5% versus 23.4%, *P* = 0.001). Furthermore, 6% of the students believed that all patients with epilepsy have the same symptoms, with a significant difference between preclinical and clinical students, with 8.8% believing that the symptoms are the same compared to 4.4% of the preclinical students (*P* = 0.000). Furthermore, when asked if epilepsy can attack at any age, participants responded yes, no, and I do not know (28.7%, 32.4%, 38.9%), respectively. Clinical students refused to believe that epilepsy could strike at any age (36.6% versus 29.3%, *P* = 0.000). Furthermore, 34.0% of the students did not think that epilepsy could be treated, 36.6% thought that it had a treatment, and 29.4% reported not knowing. Taking into account the difference between preclinical and clinical students, clinical students believed that epilepsy could be treated (49.6% versus 27.1%, *P* = 0.000) (Table 2).

**Table 2.** Response to the questions regarding general knowledge of epilepsy among medical students (yes and no answers)

	Total			Preclinical			Clinical			*P*-value
	Yes (%)	No (%)	I do not know (%)	Yes (%)	No (%)	I do not know (%)	Yes (%)	No (%)	I do not know (%)	
Heard of or read about the disease called “epilepsy” or “convulsive seizures	95.0	3.7	1.3	92.0	5.9	2.1	99.0	0.7	0.2	0.00*
Did you know anyone who had epilepsy?	38.2	58.1	3.6	38.2	56.9	4.9	38.2	59.9	1.9	0.042*
Would you object to having any of your children play with persons who sometimes had seizures (fits)?	27.7	55.3	17.0	23.4	57.1	19.5	33.5	53.0	13.5	0.001*
Would you object to having a son or daughter of yours marry a person who sometimes had seizures?	23.8	42.0	34.2	20.9	42.2	36.8	27.8	41.6	30.6	0.023*
Do you think people with epilepsy can be employed in jobs like others?	76.0	11.7	12.4	75.0	11.7	13.3	77.2	11.6	11.2	0.602
Do all patients with epilepsy have the same symptoms?	6.0	71.4	22.5	4.4	63.2	32.5	8.3	82.7	9.0	0.00*
Does epilepsy strike at any particular age?	28.7	32.4	38.9	20.4	29.3	50.3	39.9	36.6	23.5	0.00*
Is epilepsy a disease that can be treated?	36.6	34.0	29.4	27.1	33.5	39.4	49.6	34.7	15.7	0.00*

* Statistical significance.

**Figure 1.**
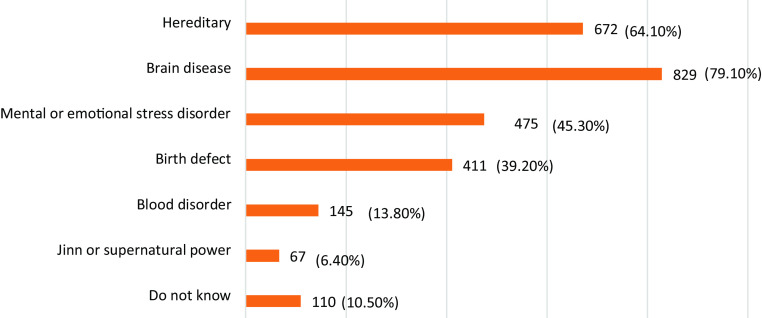
Response to the questions about causes of epilepsy among medical students (more than one answer)

In our sample size, awareness of epileptic etiologies varied between years of medical school. Most (79.1%) believe that brain disease is the cause of epilepsy, followed by hereditary causes, mental or emotional stress disorder, birth disorder, and blood disorder (64.1%, 45.3%, 39.2%, 13.8%), respectively. Furthermore, 110 participants (10.5%) stated that they were unaware of the cause of epilepsy (Figure 1).

Furthermore, we found that clinical students believed that epilepsy was an inherited condition (69.6% versus 61.4%, *P* = 0.008), caused by brain problems (86.4% versus 73.5%, *P* = 0.000) or a blood disorder (16.9% versus 11.3%, *P* = 0.012). However, no significant differences were observed between preclinical and clinical students in their knowledge of the possibility of emotional or mental stress disorders causing epilepsy, with 45.9% of preclinical students and 45.8% of clinical students believing that blood disorders are the cause of epilepsy (*P* = 0.986) (Table 3).

**Table 3.** Difference between preclinical and clinical students in their awareness about the cause of epilepsy

		Preclinical (%)	Clinical (%)	*P*-value
Causes of epilepsy	Hereditary	352 (61.4)	293 (69.6)	0.008*
	Brain disease	421 (73.5)	364 (86.4)	0.000*
	Mental or emotional stress disorder	263 (45.9)	193 (45.8)	0.986
	Birth defect	226 (39.4)	159 (37.8)	0.592
	Blood disorder	65 (11.3)	71 (16.9)	0.012*
	Jinn or supernatural power	38 (6.6)	20 (4.8)	0.211
	Do not know	85 (14.8)	20 (4.8)	0.000*

* Statistical significance.

**Figure 2.**
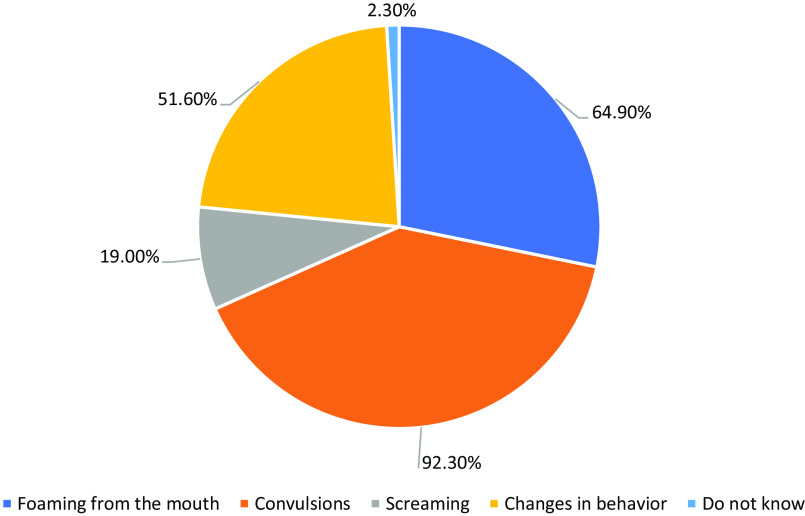
Participants’ awareness toward symptoms of epilepsy

Surprisingly, the most common symptom reported by the students was convulsion (92.3%), followed by foaming in the mouth (64.9%) and change of behavior (51.6%), with 2.3% claiming no knowledge (Figure 2).

## Social aspects

Regarding the ability of epileptic patients to have children, most of the participants (78.5%) believe that it is possible. In contrast, 7.5% believe it is not conceivable and the remaining 14% have no knowledge. In a comparison of preclinical and clinical students, preclinical students reported a higher level of ignorance (19.2% versus 6.9%), and a lower percentage of preclinical students reported the ability of an epileptic person to have a child (73.6% versus 85.0%) (*P* = 0.00). Furthermore, 21.8% of all students believed that a person with epilepsy could live alone with assistance, with no significant difference between preclinical and clinical students (*P* = 0.209). Furthermore, we found that 89.6% of general students thought it acceptable to work with epileptic people, with no significant differences between preclinical and clinical students (*P* = 0.088). Furthermore, 17.5% of the students thought that epileptic patients should be prevented from participating in sports activities, with a significant difference between preclinical and clinical students (14.3% versus 21.9%, *P* = 0.000). Furthermore, only 18% of the students believed that epilepsy affects the relatively small population of Saudi Arabia, while a higher proportion of preclinical students believed this (18.8% versus 16.9%, *P* = 0.000). Furthermore, we discovered that 41.3%, including 52.5% of preclinical students and 26.1% of clinical students, did not have information on whether epileptic patients should use antiepileptic drugs. Furthermore, 46.5% of general students believed that a role in surgical intervention is essential for advanced epileptic conditions, especially among clinical students. Finally, 46.3% of the students reported having access to any information on the treatment of epilepsy during their professional training, especially among clinical students (68.4% versus 30.0%, *P* = 0.000) (Table 4).

**Table 4.** Response to the questions regarding attitude toward epilepsy among medical students (yes and no answers)

	Total			Preclinical			Clinical			*P*-value
	Yes (%)	No (%)	I do not know (%)	Yes (%)	No (%)	I do not know (%)	Yes (%)	No (%)	I do not know (%)	
A person with epilepsy could have a child	78.5	7.5	14.0	73.6	7.2	19.2	85.0	8.1	6.9	0.00*
Person with epilepsy can live alone	21.8	51.2	27.0	20.6	50.4	29.0	23.5	52.3	24.2	0.209
It is acceptable to work with a person with epilepsy (colleague) at work	89.6	3.4	6.9	89.4	2.6	8.0	90.0	4.5	5.5	0.088
Student with epilepsy should be prevented from participating in sport activities	17.5	51.3	31.2	14.3	48.3	37.3	21.9	55.3	22.8	0.00*
In developing countries like Saudi Arabia, epilepsy affects a smaller number of people	18.0	24.9	57.0	18.8	19.4	61.8	16.9	32.5	50.6	0.00*
Every person with epileptic seizure must use an antiepileptic drug	30.4	28.3	41.3	25.5	22.0	52.5	37.1	36.8	26.1	0.00*
Is there a role for surgical intervention for advanced epileptic cases?	46.5	7.9	45.6	35.6	6.6	57.8	61.3	9.7	29.0	0.00*
Did you have access to any information of how to deal with epilepsy during your professional training	46.3	53.7	0.0	30.0	70.0	0.0	68.4	53.7	0.0	0.00*

* Statistical significance.

## Treatment

**Figure 3.**
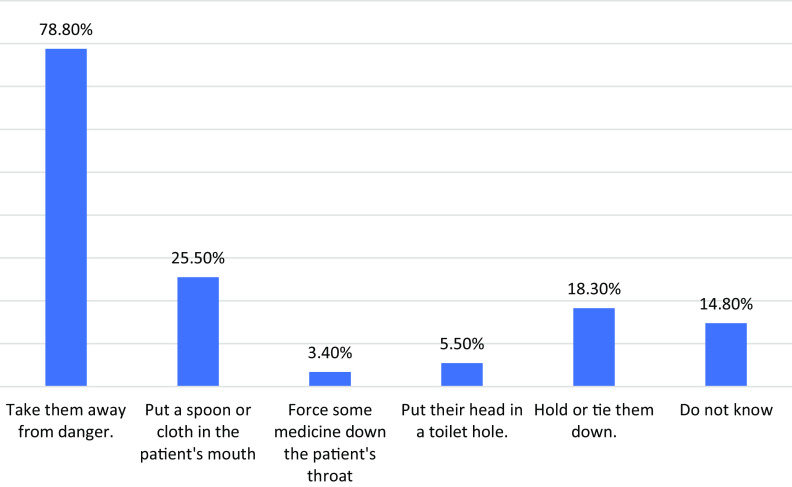
Students’ attitude toward dealing with patients with epilepsy

Taking into account the attitudes of the students toward dealing with epilepsy patients, removing them from danger was the priority of 78.8% of the students, followed by placing a spoon or cloth in the mouth (25.5%), and holding or tying (18.3%). In comparison, 14.8% did not know how to deal with patients with seizures (Figure 3).

**Figure 4.**
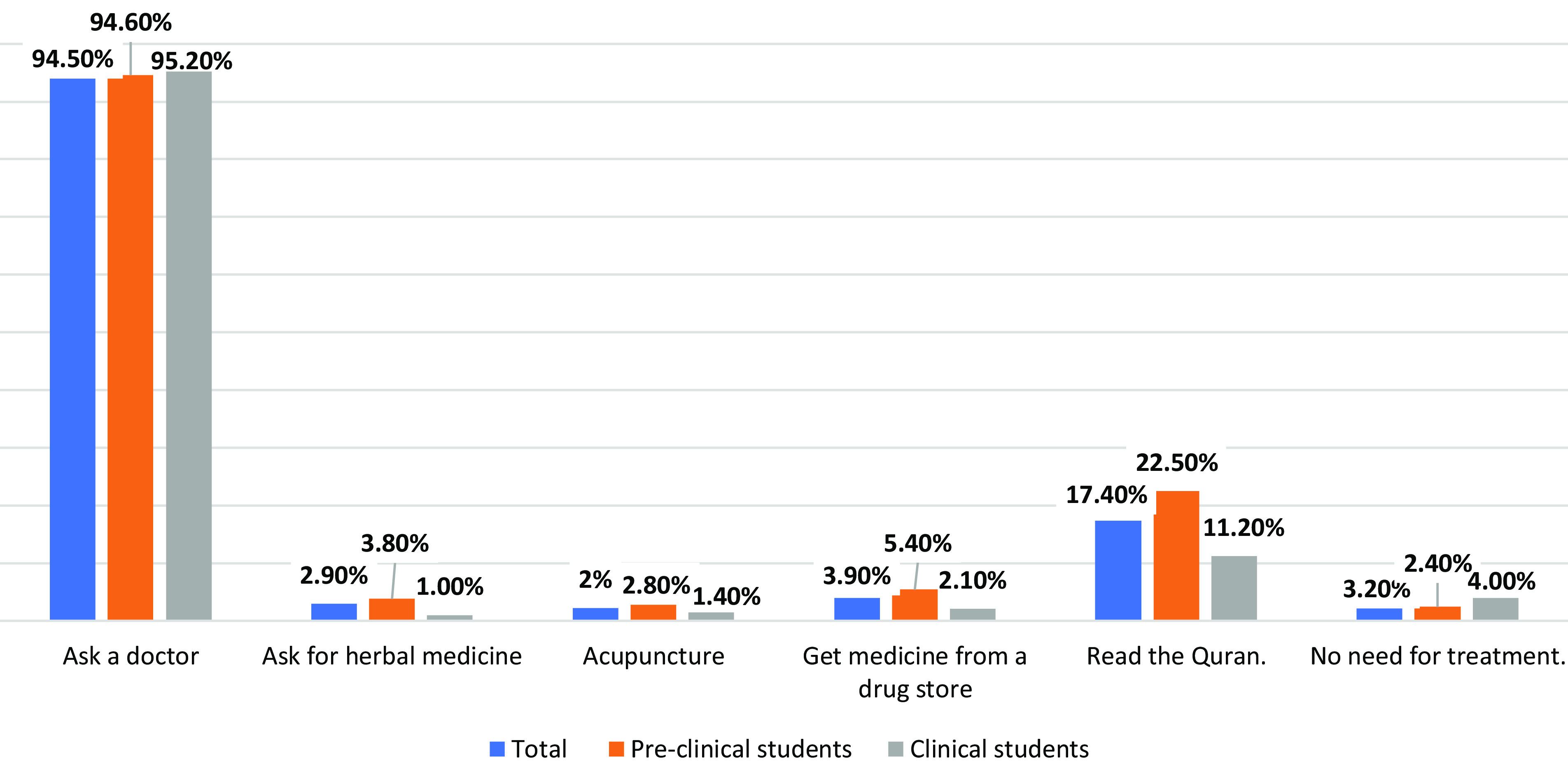
Students’ attitude toward possible treatment for epileptic patients

Taking into account possible treatment for epileptic patients, we found that most of the participants would ask for doctors without a significant difference between preclinical and clinical students (*P* = 0.641). Reading the Quran was a suggested solution for epileptic seizures where 22.5% of preclinical and 11.2% of clinical students thought reading the Quran reduces the term of epilepsy (*P* = 0.000) (Figure 4).

Furthermore, we found that 88.1% of participants would follow the instructions to avoid driving if they had epilepsy. In comparison, the main causes of not following physicians’ instructions included low public awareness of 43.8%, social reasons (37.9%), and financial reasons (21.3%) (Table 5).

**Table 5.** Participants’ attitude to follow physicians’ instruction

		Frequency	Percent
If you had epilepsy and the physician instructed you not to drive, would you abide by his instruction?	Yes	923	88.1
	No	125	11.9
Causes of not following physicians’ indications	Public awareness	94	43.8
	Social reasons	82	37.9
	Financial reasons	46	21.3
	I do not see any reason to prevent patients with epilepsy from driving.	18	8.3

## Discussion

This study’s objectives included assessing the knowledge and attitude of medical students in Riyadh city, Saudi Arabia, toward the disease of epilepsy and comparing preclinical and clinical students toward this knowledge. In this study, we included a large-scale sample of 1039 medical students from four universities in Riyadh, Saudi Arabia. The study revealed that 95% of the students reported hearing about epilepsy or convulsive seizures, especially among clinical students compared to preclinical students. This is similar to the study conducted in the Jeddah region of Saudi Arabia among medical students and students. The authors found that 95.3% of general students reported hearing about epilepsy.

In comparison, the percentage of clinical students who reported awareness of epilepsy was significantly higher than that of preclinical students (98.1% versus 89.8%, *P* = 0.005) (Alomar *et al.* 2020). However, the percentage reported in the present study is lower than that published in a previous study by Alaqeel among healthcare providers, in which 100% of the participants had heard of epilepsy (Alaqeel *et al.* 2013). However, the results of the previous study did not differ significantly from ours, since it included healthcare professionals rather than intern students. On the contrary, our investigation showed that 99% of the intern students were aware of epilepsy. Furthermore, in another study conducted among medical students at KAU, Saudi Arabia, the results showed that 95.4% of medical students of KAU are aware of the meaning of epilepsy, read or heard about it, or simply know someone with epilepsy (Shihata *et al.* 2021). This finding was better than previous studies conducted among medical students in other developing countries, including Turkey (Kartal, 2016), Brazil (Fonseca *et al.* 2004), and Malaysia (Ab Rahman, 2005). Although the Saudi community has many incorrect beliefs and prejudices about epilepsy (Obeid *et al.* 2012), the awareness of medical students revealed in this study is much better than in many other studies. It is comparable to the public awareness reported in previous studies in Saudi Arabia (Haneef *et al.* 2014; Alqurashi *et al.* 2018; Alharthi *et al.* 2019).

In this study, 6% of general medical students, particularly clinical students, reported that all epilepsy patients had the same symptoms, which is higher than the 4.3% reported in Alomar’s study (Alomar *et al.* 2020) and the 3% found in another study by Alaqeel (Alaqeel *et al.* 2013). However, this is lower than previous research on healthcare providers (Fonseca *et al.* 2004) and the general public (Alaqeel *et al.* 2015; Osama *et al.* 2016). Furthermore, convulsions (92.3%), mouth foaming (64.9%), and behavioral problems were the symptoms most commonly reported by students (51.6%) in terms of epilepsy etiologies. This finding is similar to Alomar’s findings (Alomar *et al.* 2020). However, it is worse than the previously reported study among healthcare professionals, which differs from our study in the targeted group (Alaqeel *et al.* 2013). Furthermore, we observed that 28.7% knew that epilepsy could strike at any age and 36.6% believed epilepsy could be treated. According to Alaqeel’s study, 82.3% thought epilepsy was a treatable disorder and 36.1% believed epilepsy could strike at any age (Alaqeel *et al.* 2013). We discovered that brain disease, inherited causes, and mental or emotional stress disorder were the most common causes of epilepsy according to the knowledge of the causes. According to Almori’s study, 70.3% believe that tumors cause epilepsy, followed by brain damage (67.6%), stroke (48.8%), metabolic diseases (44.5%), and insanity (30.9%) (Alomar *et al.*, 2020). According to the Alaqeel study, the most common causes of seizures reported by the participants were brain disease (95.3%), hereditary (89%), and mental and emotional disorders (27.5%) (Alaqeel *et al.* 2013), which is consistent with the findings of the current study. Furthermore, according to several studies (Fonseca *et al.*, 2004; Al-Rashed *et al.*, 2009; Panda *et al.*, 2011; Hijazeen *et al.*, 2014; Kartal, 2016), many medical students believed that epilepsy was a genetic disorder, which is consistent with the findings of the current study.

Most of the survey participants were aware of epilepsy and had favorable opinions about people with epilepsy, although some were concerned about patients with epilepsy and their professional lives. In the present study, 89.6% agreed to work with epileptic patients; however, 17.5% recommended that patients refrain from participating in sports activities, 27.3% would object to their son or daughter playing with patients with epilepsy, and 23.8% refused to marry a patient with epilepsy. This could be explained by parents’ fear of having epileptic seizures in front of their children that could injure them. However, we noticed that this unfavorable attitude was significantly lower in clinical students than in preclinical students, implying that medical school clinical courses could reduce negative attitudes toward patients with epilepsy.

Moving patients out of danger was the top priority for 78.8% of medical students, followed by inserting a spoon or rags into the patients’ mouths (25.5%). However, the Alaqeel study discovered that 98% of volunteers would remove them from danger and 67.2% would shove a spoon or cloth in the patient’s mouth (Alaqeel *et al.* 2013). However, most of the participants said they would seek medical assistance if a friend had epilepsy, while 17.4% said they would read the Quran. The reading of the Quran may be explained by the widely held belief among Saudis that the Jinn’s possession or spiritual power is one of the leading causes of epilepsy (Alaqeel *et al.* 2013; Alomar *et al.* 2020). This investigation does have certain limitations. One of these drawbacks is the reliance on self-reported questionnaires, which include some personal bias. Furthermore, the questionnaire was sent over the Internet, which resulted in some sampling bias. On the other hand, this study could be considered to be the first large-scale study conducted in Saudi Arabia’s Riyadh city.

## Conclusions

The awareness of epilepsy is better than reported in other studies, particularly among clinical students rather than preclinical students, who have a negative attitude toward epileptic patients. Consequently, there is a need to further development of your knowledge in future campaigns, conferences, and curricula that should be tailored to help improve awareness and attitudes about epilepsy.
